# Supplementary material for: Association Between the Information Environment, Knowledge, Perceived Lack of Information, and Uptake of the HPV Vaccine in Female and Male Undergraduate Students in Belgrade, Serbia
Source: Eur J Investig Health Psychol Educ. 2025 Feb 7;15(2):21. doi: 10.3390/ejihpe15020021 (PMC11854145; doi:10.3390/ejihpe15020021)
Supplement: Supplementary file 1 [file ejihpe-15-00021-s001.zip › ejihpe-3332052-supplementary.pdf]

Supplementary Material 1. Univariate and multivariate logistic regressions exploring the associations between the knowledge score and perceived lack of information with the use of sources of information

Supplementary Table 1 shows univariable and multivariable linear regression exploring the association between the knowledge score and use of sources of information about HPV vaccines. The use of scientific literature was positively associated with a higher knowledge score (β=0.40, 95% CI: 0.30 to 0.51, p<0.001), and this association remained strong and even increased in the multivariable analysis (β=0.54, 95% CI: 0.44 to 0.64, p<0.001). Internet portals were also positively associated with knowledge, both in the univariable (β=0.14, 95% CI: 0.02 to 0.25, p=0.024) and multivariable analyses (β=0.21, 95% CI: 0.09 to 0.33, p<0.001). Both national and regional TV channels were negatively associated with knowledge in the univariable analysis, with coefficients of -0.27 (95% CI: -0.39 to -0.15, p<0.001) and -0.31 (95% CI: -0.44 to -0.19, p<0.001), respectively. The negative association for national TV channels persisted in the multivariable analysis (β=-0.20, 95% CI: -0.39 to -0.02, p=0.033). Government as the source of information was negatively associated with knowledge in univariable analysis (β=-0.34, 95% CI: -0.49 to -0.19, p < 0.001). Religious leaders as a source of information were associated with lower knowledge scores in both the univariable (β=-0.63, 95% CI: -0.81 to -0.46, p<0.001) and multivariable analyses (β=-0.60, 95% CI: -0.81 to -0.39, p<0.001). Other sources such as YouTube, social networks, family, friends, and healthcare workers in the media did not show significant associations with knowledge scores in the multivariable model.

Supplementary Table 1. Univariable and multivariable linear regression exploring the association between the knowledge score and use of sources of information about HPV vaccines

**Dependent:** ***Knowledge score***	**Coefficient (univariable)**	**Coefficient (multivariable)**
Scientific literature	0.40 (0.30 to 0.51, p<0.001)	0.54 (0.44 to 0.64, p<0.001)
National TV channels	-0.27 (-0.39 to -0.15, p<0.001)	-0.20 (-0.39 to -0.02, p=0.033)
Regional TV channels	-0.31 (-0.44 to -0.19, p<0.001)	-0.13 (-0.32 to 0.06, p=0.182)
Internet portals	0.14 (0.02 to 0.25, p=0.024)	0.21 (0.09 to 0.33, p<0.001)
Youtube	0.04 (-0.07 to 0.15, p=0.482)	-
Social Networks	0.06 (-0.05 to 0.17, p=0.266)	-
Family	-0.09 (-0.20 to 0.01, p=0.089)	0.00 (-0.10 to 0.10, p=0.952)
Friends	0.04 (-0.08 to 0.17, p=0.503)	-
My doctor	0.03 (-0.07 to 0.14, p=0.525)	-
Healthcare workers in media	0.04 (-0.07 to 0.15, p=0.494)	-
Religious leaders	-0.63 (-0.81 to -0.46, p<0.001)	-0.60 (-0.81 to -0.39, p<0.001)
Government	-0.34 (-0.49 to -0.19, p<0.001)	-0.16 (-0.34 to 0.02, p=0.085)

Supplementary Table 2 shows the univariable and multivariable linear regression analyses examining the association between perceived lack of information about HPV vaccines and use various sources of information. In the univariable analysis, using scientific literature as a source of information was significantly associated with a lower perceived lack of information (β=-0.18, 95% CI: -0.24 to -0.11, p<0.001), and the negative association was stronger in the multivariable analysis (β=-0.21, 95% CI: -0.27 to -0.14, p<0.001). The influence of religious leaders was positively associated with a higher perceived lack of information in both the univariable (β=0.20, 95% CI: 0.09 to 0.31, p<0.001) and multivariable analyses (β=0.22, 95% CI: 0.08 to 0.35, p=0.002). The use of regional TV channels also showed a positive association with perceived lack of information in the univariable analysis (β=0.10, 95% CI: 0.03 to 0.18, p=0.006), and a marginally significant effect in the multivariable model (β=0.12, 95% CI: -0.00 to 0.24, p=0.050). Getting information from a personal doctor was associated with a lower perceived lack of information in both the univariable (β=-0.08, 95% CI: -0.14 to -0.02, p=0.014) and multivariable analyses (β=-0.07, 95% CI: -0.14 to -0.01, p=0.022). Other sources, such as national TV channels, internet portals, and social networks, did not show a significant association with perceived lack of information in the multivariable analysis.

Supplementary Table 2. Univariable and multivariable linear regression exploring the association between perceived lack of information and sources of information about HPV vaccines

**Dependent:** ***Perceived lack of information***	**Coefficient (univariable)**	**Coefficient (multivariable)**
Scientific literature	-0.18 (-0.24 to -0.11, p<0.001)	-0.21 (-0.27 to -0.14, p<0.001)
National TV channels	0.06 (-0.01 to 0.14, p=0.090)	-0.00 (-0.12 to 0.12, p=0.946)
Regional TV channels	0.10 (0.03 to 0.18, p=0.006)	0.12 (-0.00 to 0.24, p=0.050)
Internet portals	-0.06 (-0.12 to 0.01, p=0.116)	-0.05 (-0.13 to 0.02, p=0.153)
YouTube	0.04 (-0.03 to 0.10, p=0.289)	-
Social Networks	-0.01 (-0.07 to 0.06, p=0.811)	-
Family	-0.00 (-0.06 to 0.06, p=0.994)	-
Friends	-0.04 (-0.11 to 0.03, p=0.256)	-
My doctor	-0.08 (-0.14 to -0.02, p=0.014)	-0.07 (-0.14 to -0.01, p=0.022)
Healthcare workers in media	-0.03 (-0.10 to 0.04, p=0.362)	-
Religious leaders	0.20 (0.09 to 0.31, p<0.001)	0.22 (0.08 to 0.35, p=0.002)
Government	0.09 (0.00 to 0.18, p=0.049)	0.05 (-0.07 to 0.16, p=0.418)
